# Efficacy and Safety of Combined Autologous Blood and Minocycline Pleurodesis for Intractable Pneumothorax in High‐Risk Non‐Surgical Patients: A Case Series

**DOI:** 10.1002/rcr2.70430

**Published:** 2025-12-08

**Authors:** Eitetsu Koh, Yasuo Sekine

**Affiliations:** ^1^ Department of Thoracic Surgery Tokyo Women's Medical University Yachiyo Medical Center Chiba Japan

**Keywords:** autologous blood pleurodesis, interstitial lung disease, minocycline pleurodesis, pneumothorax

## Abstract

Pneumothorax in patients with interstitial lung disease (ILD), severe COPD, or other significant comorbidities poses a serious therapeutic challenge, especially when surgery is contraindicated. We report a case series of 22 high‐risk patients with intractable secondary spontaneous pneumothorax (SSP) managed with combined autologous blood and minocycline pleurodesis. This bedside, sequential and non‐mixed protocol terminated persistent air leak without major complications or recurrence during a 6‐month follow‐up. Our findings suggest that this simple and accessible approach may serve as a valuable non‐surgical alternative in medically inoperable patients.

## Graphical abstract

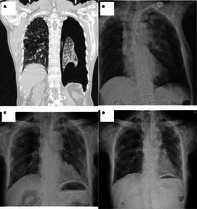

## 1 Introduction

Spontaneous pneumothorax complicating ILD or severe COPD can be life‐threatening and difficult to manage when surgical options are precluded by limited pulmonary reserve or comorbidities [1, 2]. Pleurodesis using autologous blood (ABP) or minocycline has been individually reported as effective for persistent air leak [3, 4, 5, 6, 7], yet evidence on their combined use—particularly in nonsurgical, high‐risk SSP—remains limited. We adopted a pragmatic, sequential, non‐mixed bedside protocol combining minocycline with ABP and describe our centre's experience, in the context of guideline‐based management of SSP and pleural disease [1, 2, 8, 9].

## 2 Case Series

We retrospectively reviewed 22 consecutive high‐risk patients with intractable SSP managed at our institution between 2022 and 2024. Intractable SSP was defined as a continuous or inducible air leak persisting for ≥ 7 days despite standard chest‐tube drainage in non‐surgical candidates. Cessation of air leak was defined as ≥ 24 h absence of bubbling on water seal at rest and with provoked cough, with no radiographic re‐collapse before tube removal. Pulmonary function tests were not performed because patients presented with pneumothorax (acute state).

After confirming adequate lung re‐expansion on water seal, minocycline 100 mg diluted in 50 mL normal saline was instilled first, followed by 50 mL autologous blood via a separate syringe. Agents were not mixed. A 10–20 mL saline flush was given. The tube was not clamped and was kept elevated on water seal. Low suction (−10 to −20 cm H_2_O) was considered 2–4 h later only if clinically required. Sessions were repeated every 48–72 h if the leak persisted.

A 20‐Fr large‐bore chest catheter was used in all patients.

Twenty‐two patients were included. The mean age was 74 years (range 63–88), and 21/22 (95%) were male. Underlying conditions included severe COPD (*n* = 11), interstitial lung disease (ILD; *n* = 8), COPD–ILD overlap (*n* = 1), congestive heart failure (*n* = 1) and bronchiectasis (*n* = 1). All patients were judged non‐surgical candidates by a multidisciplinary team owing to limited pulmonary reserve and/or comorbidities. Pulmonary function tests were not performed at presentation due to the pneumothorax.

Baseline characteristics and outcomes are summarised in Table 1.

**TABLE 1:** Demographic and clinical characteristics of the study population.

Variable	Value
Number of patients	22
Age, mean (range)	74 (63–88) years
Sex, male	21 (95%)
Underlying condition	
Severe COPD	11 (50%)
ILD (including IPF and CVD‐ILD)	8 (36%)
COPD + ILD overlap	1 (5%)
Congestive heart failure	1 (5%)
Bronchiectasis	1 (5%)
Long‐term corticosteroid use	7 (32%)
Number of pleurodesis sessions, mean (range)	2.9 (1–7)
Adverse events	
Low‐grade fever	1 (5%)
Empyema	0
Other complications	0
Mortality during admission	1 (due to comorbidity, not pleurodesis)
Recurrence at 6‐month follow‐up	0

Abbreviations: COPD, chronic obstructive pulmonary disease; ILD, interstitial lung disease; IPF, D‐ILD, collagen vascular disease‐associated ILD.

The mean number of pleurodesis sessions was 2.9 (median 3; range 1–7). Successful cessation of air leak—defined above—was achieved in (22/22 [100%]). Time from chest‐tube insertion to first pleurodesis was (median 5 days [IQR 2–12]), and time to air‐leak cessation was (median 5 days [IQR 2–12]) after the first instillation. Chest‐tube duration was (median 7 days [IQR 3–15]), and length of stay was (median 8 days [IQR 3–20]).

**FIGURE 1:**
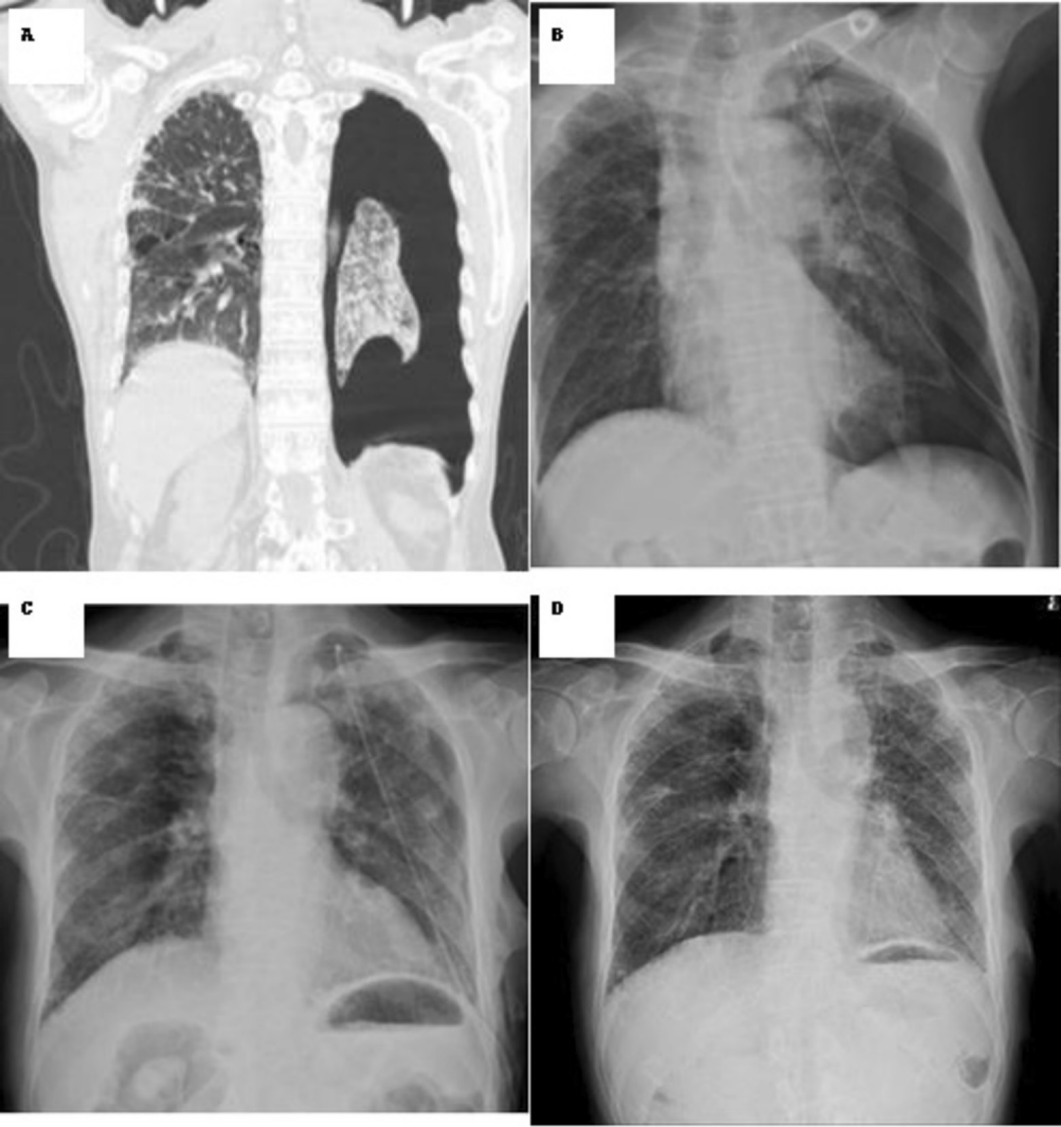
Representative case and radiologic course (panels A–D). Air‐leak cessation was confirmed by ≥ 24 h absence of bubbling on water seal at rest and with provoked cough, with stable chest radiography prior to tube removal. Pulmonary function tests were not performed owing to pneumothorax.

One patient developed a transient low‐grade fever; no empyema or other significant complications occurred. In one patient with prior unsuccessful pleurodesis using OK‐432, death due to respiratory failure occurred after air‐leak resolution. No recurrences were observed during 6‐month follow‐up. A representative radiologic course is shown in Figure 1 (panels A–D).

## 3 Discussion

This case series suggests that sequential, non‐mixed autologous blood plus minocycline pleurodesis is a feasible option for intractable secondary spontaneous pneumothorax (SSP) in high‐risk, nonsurgical patients, many of whom had ILD and/or severe COPD. We deliberately used a pragmatic bedside protocol (minocycline first, autologous blood second; no clamping; water‐seal; optional low suction) to balance effectiveness, safety and reproducibility in a frail population in whom escalation to surgery or general anaesthesia was undesirable [1, 2, 8, 9].

The rationale for combining modalities was that mechanistically, autologous blood provides an immediate ‘patch’ effect—fibrin deposition and clot formation around the fistula and along the pleural surface—while minocycline generates a chemical pleuritis that promotes durable pleurodesis. Either agent alone has been used for persistent air leak; our intent was to leverage rapid leak control (blood patch) and longer‐term pleural symphysis (minocycline) in one standardised sequence, while avoiding the potential for clot dilution or drug inactivation that might occur if mixed in the same syringe [3, 5, 6, 7, 8]. This conceptual pairing is attractive in SSP with fragile parenchyma, where spontaneous sealing is slow and the risk of prolonged drainage is high.

The reason why we did not use talc in this cohort is that although talc is effective for pleurodesis [10, 11], in elderly patients with ILD/COPD, we prioritised a regimen we considered lower in systemic inflammatory burden and easier to deliver at the bedside. While high‐quality comparative trials in this exact population are lacking, clinical judgement favoured avoiding talc where diffuse pleural inflammation or acute lung injury could theoretically worsen gas exchange in impaired reserve. Our results—no empyema, only transient low‐grade fever and no early recurrence—support that the safety profile of the current approach was acceptable for this comorbidity‐burdened group.

The following should be considered for standardisation of the procedural details. Several elements likely contributed to outcomes: (i) requiring adequate re‐expansion on water‐seal before instillation; (ii) sequential—not mixed—delivery (minocycline 100 mg/50 mL followed by autologous blood 50 mL) with a 10–20 mL saline flush; (iii) no clamping, to avoid tension physiology and migration of agents into large airways; and (iv) delayed, low‐level suction (−10 to −20 cm H_2_O) only if clinically needed after 2–4 h [1, 2, 8, 9, 12]. We also used a 20‐Fr large‐bore chest catheter in all cases, which may balance drainage efficiency and tube patency during blood instillation, reducing the risk of lumen occlusion relative to small‐bore drains.

An important point is the definition of ‘intractable’ and leak cessation and their clinical relevance. In SSP, ongoing fistula flow and alveolar‐pleural pressure gradients are typically higher than in PSP. We therefore selected a ≥ 7‐day threshold for intractability (post‐drainage) to identify truly persistent leaks unlikely to resolve promptly without pleurodesis. For cessation, we required ≥ 24 h absence of bubbling on water‐seal at rest and with provoked cough, plus no radiographic re‐collapse before tube removal. These operational definitions reduce subjective variation and can be implemented at the bedside, facilitating replication across units [1, 2, 8, 9, 12].

A mean of 2.9 sessions (median 3) was needed, reflecting the fistula complexity and underlying parenchymal frailty in SSP/ILD/COPD. We repeated sessions every 48–72 h if bubbling persisted, allowing time for fibrin stabilisation and pleural adhesion to evolve between instillations while maintaining a predictable care pathway.

Existing reports on ABP or tetracycline‐class agents show variable success, often shaped by drain strategy, leak severity and patient selection. Our series adds procedural granularity (sequence, volumes, suction policy and re‐expansion requirement) and focuses on nonsurgical SSP—a group frequently under‐represented in trials [3, 5, 6, 7, 12]. Although single‐centre and retrospective, the approach is logistically simple, uses readily available materials and may be generalisable to resource‐constrained settings and to patients with poor anaesthetic candidacy.

We observed no empyema and only transient fever in one patient. Avoiding agent mixing, minimising suction early after instillation and not clamping may have reduced adverse events such as tube blockage, tension, or chemical bronchitis. Nonetheless, clinicians should maintain vigilance for infection, severe pain, fever and oxygenation changes—especially in ILD.

The strengths of this study include (i) a uniform protocol, (ii) clear, bedside‐applicable definitions of intractability and cessation and (iii) inclusion of high‐risk SSP patients often excluded from trials. Limitations are (i) retrospective design without a control arm (e.g., talc, ABP‐only, or minocycline‐only), (ii) potential selection and information bias, (iii) modest sample size, (iv) PFTs unavailable at presentation due to pneumothorax and (v) 6‐month follow‐up that, while clinically relevant, may miss late recurrence. These constraints temper causal inference but do not negate the consistent clinical signal we observed [8, 9, 12].

For nonsurgical SSP with ≥ 7 days of leak, a sequential, non‐mixed ABP + minocycline protocol provides a step‐wise, reproducible option before considering more invasive interventions. Prospective, comparative studies should test ABP + minocycline versus talc (or vs. single‐agent strategies) in SSP/ILD‐COPD populations, incorporate objective air‐flow measurements and evaluate patient‐centred outcomes (time to mobility, length of stay, readmission and quality of life).

## Funding

The authors have nothing to report.

## Consent

Written informed consent was obtained from all patients or their legal representatives for publication of this case series. The consent was obtained using the official ‘Respirology Case Reports Patient Consent Form’, in accordance with the journal's ethical requirements.

## Conflicts of Interest

The authors declare no conflicts of interest.

## Data Availability

The data that support the findings of this study are available from the corresponding author upon reasonable request.
